# Entrustable professional activities in Finnish radiology training: a national survey

**DOI:** 10.1186/s13244-025-01992-w

**Published:** 2025-06-05

**Authors:** Jussi Hirvonen, Suvi Marjasuo, Sanna Huhtaniska, Mervi Tiihonen, Milja Holstila

**Affiliations:** 1 Department of Radiology, University of Turku and Turku University Hospital, Turku, Finland; 2 Department of Radiology, Tampere University, Faculty of Medicine and Health Technology, Tampere University Hospital, Tampere, Finland; 3 Radiology, University of Helsinki and HUS Diagnostic Center, Helsinki University Hospital, Helsinki, Finland; 4 Research Unit of Health Sciences and Technology, Faculty of Medicine, University of Oulu, and Department of Diagnostic Radiology, Oulu University Hospital, Oulu, Finland; 5 Department of Clinical Radiology, Institute of Medicine, University of Eastern Finland, Kuopio, Finland

**Keywords:** Graduate medical education, Training program, Curriculum

## Abstract

**Objectives:**

This study assessed the practical implementation, experiences, and attitudes toward entrustable professional activities (EPAs) in radiology training across Finland.

**Methods:**

A nationwide, anonymous online survey targeted radiology residents, recently graduated specialists (within 3 years), and instructor specialists. Distributed to all Finnish hospitals involved in radiology training, the survey evaluated EPA completion rates, perceived value, and future development needs. Responses were analyzed to identify trends and differences across groups.

**Results:**

Of 150 respondents (42% residents, 43% instructors, and 14% recent graduates), 65% were from university hospitals. Among residents and recent graduates, 37% had completed EPA assessments, with 87% valuing the feedback received and 73% finding EPAs effective for competency assessment. Overall, 64% considered EPAs well-suited to radiology. Residents showed higher completion rates (43%) than recent graduates (19%), with fourth- and fifth-year residents more engaged (69% vs. 15%). Instructors, while supportive (67% viewed EPAs as meaningful), emphasized a need for more training (54% vs. 49% of residents).

**Conclusion:**

Most Finnish radiology respondents considered EPAs well-suited for training. Residents and recent graduates who completed EPAs greatly valued the feedback and found them effective for assessing competencies, with residents participating more actively than recent graduates. Instructors’ desire for better guidance suggests a priority for enhanced support and education. These findings endorse EPA integration and inform refinements in national and European radiology curricula.

**Critical relevance statement:**

Finnish radiologists and residents strongly support EPAs in radiology training, valuing their feedback and competency assessment, though instructors seek enhanced guidance.

**Key Points:**

- Finnish radiology residents and specialists reported positive experiences and strong support for entrustable professional activity (EPAs).
- Finland’s mandatory, nationally coordinated EPA framework contrasts with subspecialty-focused models elsewhere.
- Instructors seek more EPA training, signaling a need for enhanced education to sustain their engagement as adoption grows.

**Graphical Abstract:**

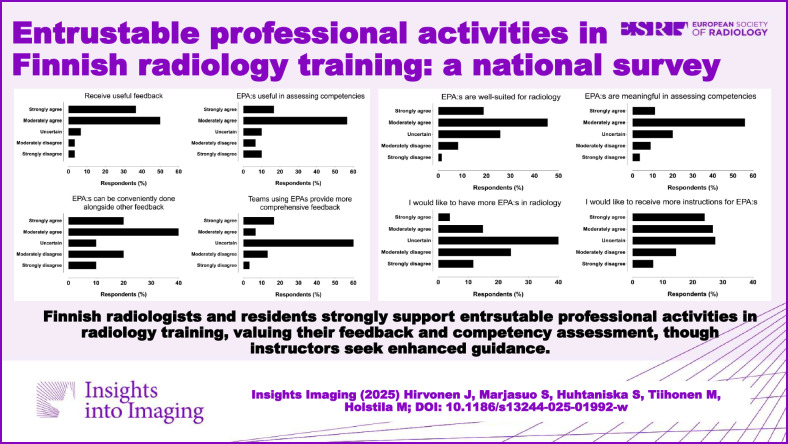

## Introduction

Medical education has increasingly shifted from traditional knowledge-based models to competency-based education (CBE), necessitating practical tools for assessing competencies. Entrustable professional activities (EPAs) have gained traction across medical specialties as a method to integrate core knowledge with broader skills. EPAs enable senior physicians to delegate tasks to residents once they demonstrate sufficient competence, offering a holistic measure of performance confidence and an opportunity for structured feedback [1, 2]. This approach has been widely adopted in the medical field [3–5].

In radiology, initial reports on EPA development and application have emerged [6–9]. Many published examples target general professional competencies [6] or subspecialty-level aims [8, 9], with less emphasis on general radiology aims [7], limiting their generalizability to a common European educational framework. A recent European Society of Radiology (ESR) survey of its national institutional member societies explored EPA use and perceptions, among other educational topics [10]. With 65 responses from 38 countries, this international survey found EPAs implemented in 21% of nations and planned in 26%, with 69% of respondents deeming them well-suited or somewhat suited for radiology training [10]. Building on this, the ESR incorporated introductory EPA guidance into the revised European Training Curriculum (ETC), a unified framework strongly recommended for European radiology residency programs [11–13].

However, detailed insights into EPA use, experiences, and attitudes within individual ESR member societies remain scarce. In Finland, the national authority mandated EPAs across all 50 medical specialties in 2021, introducing five radiology-specific EPAs for residents in 2022 and expanding to eight in 2025. This nationwide rollout positions Finland as a critical case study for evaluating EPA applicability in radiology training. Finland’s uniform curriculum, implemented across five university hospitals, ensures standardized competency assessments aligned with the ETC. This centralized approach allows for a robust evaluation of EPAs’ practical integration, offering insights into their scalability and adaptability that could inform other nations’ training frameworks. Finland’s radiology residency spans 5 years, typically split between 2–3 years at non-university hospitals and 2–3 years at one of five university hospitals (formal range: 1–4 years each). The nationally coordinated curriculum adheres to the European Training Curriculum (ETC) [11]. In addition to EPAs, there is a national logbook.

In this study, we surveyed Finnish radiologists and residents to evaluate the current national landscape and help plan future EPA development. Based on the previous international survey and discussions among national radiology educators, we expected that EPAs would be well-introduced and considered suitable for Finnish radiology training.

## Methods

### Study design

This was a prospective survey study targeting radiology residents, specialist instructors, and recently graduated (within the past 3 years) specialists on the topic of experience and attitudes toward EPAs in radiology. Per Finnish legislation, this anonymous expert opinion survey required neither ethics committee approval nor informed consent.

### Sampling and sample size

We targeted physicians in the field of radiology in all Finnish hospitals, public and private. As of 2021, Finland had 667 radiology specialists and 160 residents. We did not perform sample size estimations for statistical power. An anonymous online survey (Appendix A), developed by the Radiological Society of Finland’s Education Committee, was emailed to all Finnish hospitals involved in radiology resident training on February 3, 2025. A reminder was sent on February 10, and the study was closed on February 14, 2025.

### Inclusion and exclusion criteria

We included all responses as the survey was completely anonymous, and the data could not be confirmed. Missing data were ignored. There were no exclusion criteria.

### Survey development

The survey (Appendix A) was developed by the Radiological Society of Finland’s Education Committee. It contained brief background questions on group status and, in the case of residents, the year and university of residency training. Questions on the experiences and attitudes were assessed using five-level Likert scales, where 1 = strongly disagree, 2 = moderately disagree, 3 = uncertain, 4 = moderately agree, and 5 = strongly agree. Additional questions were multiple choice or free text. The survey was not piloted or formally validated before deployment due to its exploratory nature.

### Statistical analyses

Responses were analyzed and reported as percentages of total respondents. We used descriptive statistics (means, standard deviations, percentages) to summarize the results. Univariate associations were assessed with Chi-squared tests for nominal variables and t-tests for continuous variables, conducted using IBM SPSS Statistics for Mac (version 28, IBM Corporation, 2021). Statistical significance was set at *p* < 0.05.

## Results

### Respondent characteristics and completion of EPA assessments

We received 150 responses, 65% from university hospitals and 35% from other hospitals involved in radiology training. Of 149 respondents with complete data, 63 (42%) were residents, 65 (44%) were specialist instructors, and 21 (14%) were specialists who graduated within the past 3 years, yielding approximate response rates of 39% (residents) and 13% (specialists).

About one-third (37%) of the residents and recently graduated specialists had completed EPA assessments. Current residents (43%) showed a trend toward higher completion rates than recently graduated specialists (19%; *p* = 0.053), with a mean of 2.3 EPAs completed (range 1–5). As anticipated, fourth- and fifth-year residents reported higher completion (69%) than first- and second-year residents (15%; *p* = 0.015), with no significant differences across university versus non-university hospitals (*p* = 0.711) or among affiliate universities (*p* = 0.541).

### EPA assessment experiences

**Fig. 1:**
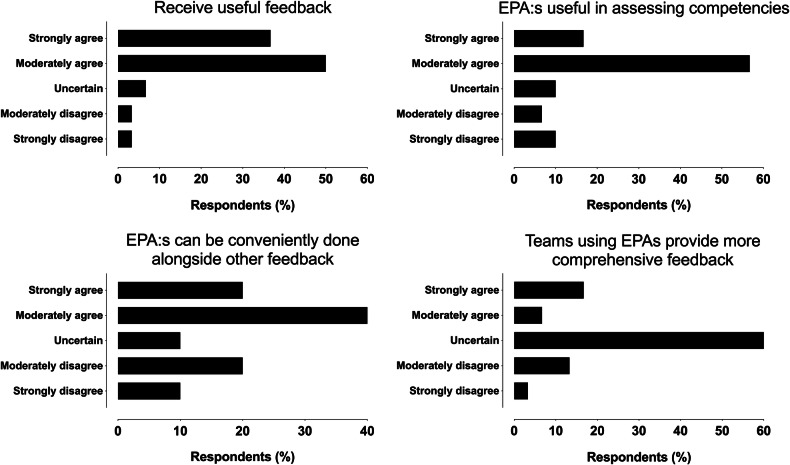
Response distributions of reported experiences from those residents and recently graduated specialists who had completed EPA assessments, with regard to receiving useful feedback (top left), usefulness of EPAs in assessing competencies (top right), convenience in completing EPAs alongside other feedback (bottom left), and whether feedback is more comprehensive in teams that use EPAs (bottom right)

Among those who completed EPAs, feedback was largely positive: 87% strongly or moderately agreed they received useful feedback, 73% found EPAs valuable for assessing competencies, and 60% rated them convenient alongside other feedback mechanisms (Fig. 1). Views on whether subspecialty teams using EPAs provided more comprehensive feedback were mixed, with 60% neutral (Fig. 1). Experiences were not statistically significantly associated with the year of residency.

### Attitudes toward EPAs and wishes for future developments

**Fig. 2:**
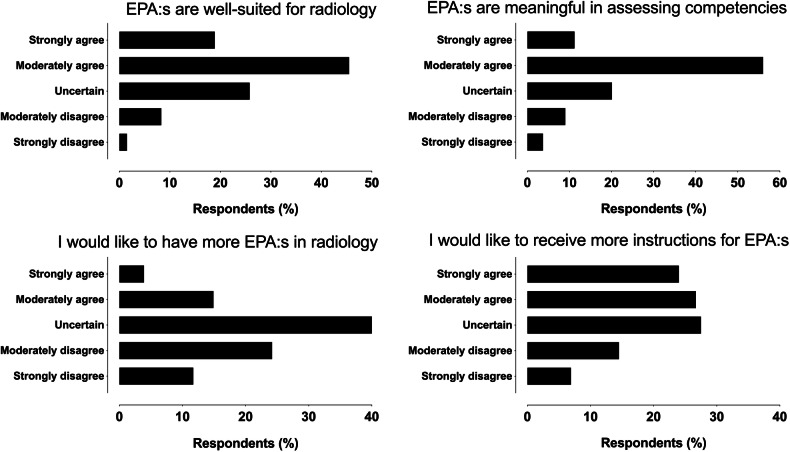
Response distributions among all respondents on the suitability of EPAs in radiology (top left), meaningfulness of EPAs in assessing competencies (top right), wanting more EPAs in radiology (bottom left), and whether they would like to receive more instructions on EPAs (bottom right)

Overall, 64% of respondents viewed EPAs as very or somewhat suited to radiology (Fig. 2), with no differences across groups (*p* = 0.364). Similarly, 67% considered EPAs a meaningful competency assessment tool, with consistent opinions across roles (*p* = 0.436) (Fig. 2). There was a statistically significant association between viewing EPAs very or somewhat suited to radiology and year of residency (*p* = 0.023), driven mainly by first- and second-year residents being uncertain in their responses.

Only 19% favored adding more EPAs, while 36% opposed it, and 45% were undecided (*p* = 0.745) (Fig. 2). Instructors (54%) were more likely than residents (49%) or recent graduates (47%) to desire additional guidance on EPA assessments (*p* = 0.038) (Fig. 2), a need echoed by 25% (12/48) of free-text responses.

## Discussion

Finland’s nationwide adoption of EPA assessments within its radiology residency curriculum offers a unique lens to evaluate their practical use, experiences, and attitudes. Drawing a representative sample from residents, recent graduates, and specialist instructors, our survey uncovered strong support for EPAs’ suitability in radiology, alongside areas needing refinement.

The structure of EPAs in Finland’s radiology residency program, mandated nationally since 2021, with five initial EPAs expanding to eight by 2025, reflects a tailored approach aligned with the European Training Curriculum (ETC) [11]. Unlike some European models where EPAs target subspecialties, such as the 13 hepatobiliary and gastrointestinal EPAs developed recently [9], Finland employs a broader competency framework, covering general imaging interpretation to procedural skills across various radiology settings. This contrasts with the United States, where EPAs often align with Accreditation Council for Graduate Medical Education (ACGME) milestones, focusing on specific tasks like breast imaging assessments [8]. Finland’s nationally coordinated rollout of EPAs ensures uniformity across its five university hospitals, likely contributing to the favorable attitudes observed in our survey. While the ETC offers exemplary EPAs [11], Finland’s mandatory, standardized approach may limit subspecialty depth compared to more flexible international frameworks, suggesting a potential area for future enhancement.

Among residents, 43% had completed at least one EPA assessment. Though this might appear low given their mandatory status, it aligns with Finnish regulations: residents are evaluated under the curriculum active at their start date, so the 2022 EPA mandate applies to only about half. Before the implementation of the current CBE-based curriculum, residency programs were solely time-based, and some existing residents remain subject to the earlier regulations. Despite this, those completing EPAs reported highly positive experiences, most valuing their utility in assessing competencies and the feedback received. These findings support ongoing EPA use in radiology.

Instructor specialists, who oversee daily EPA assessments, are pivotal to their success, yet face an added workload, often without additional compensation. Encouragingly, instructors viewed EPAs as positively as residents and recent graduates, showing consistent support across roles. However, they more frequently sought additional training on EPA implementation (54% vs. 49% and 47%, *p* = 0.038), highlighting a need for improvement. The updated ETC offers valuable EPA guidance and international examples [11], but given cross-country variations, national or institutional training is essential to sustain instructors’ motivation and optimism.

Instructors’ desire for additional training may stem from high clinical workloads, limited familiarity with EPA frameworks, or variable institutional support for faculty development. If unaddressed, these unmet needs could lead to inconsistent EPA assessments, reduced instructor engagement, or slower adoption, potentially undermining the curriculum’s effectiveness. Targeted training initiatives are thus critical to sustain the positive attitudes toward EPAs.

Several strategies could enhance their implementation and impact to build on the strong support for EPAs observed in Finnish radiology training. First, developing national workshops and integrating EPA training into faculty development programs could address the instructors’ need for guidance, mitigating challenges such as high workloads and unfamiliarity with EPA principles that may otherwise hinder consistent assessments. Second, creating digital tools, such as mobile apps for streamlined EPA documentation, could improve convenience and engagement, particularly for busy residents and specialists. Third, piloting subspecialty-specific EPAs, inspired by international models like hepatobiliary frameworks [9], could complement Finland’s general competency approach, potentially increasing subspecialty depth while maintaining uniformity. These steps could also inform European and international programs, as Finland’s standardized model offers a scalable framework for countries planning EPA adoption per ESR surveys [10]. Future research should explore these interventions’ effectiveness, assess long-term EPA outcomes on resident competency, and compare Finland’s approach with other nations to refine global radiology training curricula.

This study benefits from Finland’s nationally coordinated curriculum and mandatory assessments, supported by a strong resident response rate of 39%. The specialist response rate of 13% may raise concerns but reflects a broad denominator of all 667 radiologists based on 2021 data, many of whom do not train residents daily. However, this low response rate introduces potential selection bias, as respondents may represent instructors more engaged with or favorable toward EPAs, potentially skewing attitudes positively and limiting generalizability to less involved specialists. Similarly, while the 39% resident response rate is robust, it may not fully capture perspectives from less motivated or busier trainees, which could overstate EPA acceptance. Potential sampling and response biases restrict our findings, as does their uncertain applicability across ESR member societies, where educational practices differ despite widespread ETC adoption [10]. To address these issues, future studies could employ targeted sampling to ensure broader specialist participation, offer incentives to improve response rates, and conduct multi-country surveys to enhance generalizability across diverse training contexts. Nevertheless, these results establish a basis for understanding EPA use in European radiology, highlighting the need for additional cross-national data to gain a comprehensive view.

In conclusion, EPAs are progressively being implemented in competency assessments of radiology residency curricula in Finland. We found very good experiences among residents and generally favorable attitudes toward the EPA assessment in radiology among specialists. As the use of these assessments is becoming more prevalent, more education is needed for both residents and instructors. Similar data from other countries is needed for a comprehensive European view.

## Supplementary information

ELECTRONIC SUPPLEMENTARY MATERIAL

## Data Availability

All relevant data are within the manuscript.

## Abbreviations

CBE: Competency-based education
EPA: Entrustable professional activity
ESR: European Society of Radiology
ETC: European Training Curriculum
