# Supplementary material for: Entrustable professional activities in Finnish radiology training: a national survey
Source: Insights Imaging. 2025 Jun 5;16:118. doi: 10.1186/s13244-025-01992-w (PMC12141187; doi:10.1186/s13244-025-01992-w)
Supplement: Supplementary file 1 — ELECTRONIC SUPPLEMENTARY MATERIAL [file 13244_2025_1992_MOESM1_ESM.docx]

**Supplementary Table 1.** Online survey.

| **Questions** | **Responses** |
| --- | --- |
| Workplace | Non-university hospital |
|  | University hospital |
| Role | Resident |
|  | Instructor specialist |
|  | Recently (<3 years) graduated specialist |
| *Year of residency | [1-5] |
| *What year did you start residency? | [year] |
| *Which university are you affiliated with? | Helsinki |
|  | Tampere |
|  | Turku |
|  | Kuopio |
|  | Oulu |
|  | Not yet affiliated |
| *According to which national program are you training? | Older time-based |
|  | New competency-based |
| *I have completed EPA assessments | Yes |
|  | No |
| *Place of EPA assessments | Non-university hospital |
|  | University hospital |
|  | Not yet completed |
| *I have completed EPA assessments (times) | Only once each |
|  | Some EPAs more than once |
| *Choose all EPAs you have completed | Hosting a multidisciplinary meeting as a radiologist |
|  | Performing an ultrasound-guided procedure |
|  | Breast examination |
|  | Image interpretation, reporting, and communication with clinicians |
|  | Imaging of the acute abdomen |
|  | Special features of pediatric imaging |
|  | Imaging of acute stroke |
|  | Reviewing and presenting scientific information |
| *I receive useful feedback about my competencies during EPA assessments | Strongly disagree |
|  | Moderately disagree |
|  | Uncertain |
|  | Moderately agree |
|  | Strongly agree |
| *The feedback is more comprehensive in teams that use EPAs | Strongly disagree |
|  | Moderately disagree |
|  | Uncertain |
|  | Moderately agree |
|  | Strongly agree |
| *EPAs are useful in assessing competencies | Strongly disagree |
|  | Moderately disagree |
|  | Uncertain |
|  | Moderately agree |
|  | Strongly agree |
| EPA assessments can be conveniently done alongside other feedback | Strongly disagree |
|  | Moderately disagree |
|  | Uncertain |
|  | Moderately agree |
|  | Strongly agree |
| I would like to have more EPAs in radiology | Strongly disagree |
|  | Moderately disagree |
|  | Uncertain |
|  | Moderately agree |
|  | Strongly agree |
| EPAs are meaningful way to assess competencies of residents | Strongly disagree |
|  | Moderately disagree |
|  | Uncertain |
|  | Moderately agree |
|  | Strongly agree |
| EPAs are well suited for radiology | Strongly disagree |
|  | Moderately disagree |
|  | Uncertain |
|  | Moderately agree |
|  | Strongly agree |
| I would like to have more instruction on EPA assessments | Strongly disagree |
|  | Moderately disagree |
|  | Uncertain |
|  | Moderately agree |
|  | Strongly agree |
| Free text feedback | [text] |

*Question only for current residents and recently graduated specialists.

**Supplementary Table 2.** List of radiology EPAs currently used in Finland.

Hosting a multidisciplinary meeting as a radiologist

Performing an ultrasound-guided procedure

Breast examination

Image interpretation, reporting, and communication with clinicians

Imaging of the acute abdomen

Special features of pediatric imaging

Imaging of acute stroke

Reviewing and presenting scientific information
